# Allogeneic Transplantation in Chronic Myeloid Leukemia and the Effect of Tyrosine Kinase Inhibitors on Survival: A Quasi-Experimental Study

**DOI:** 10.4274/tjh.2015.0346

**Published:** 2017-03-01

**Authors:** Mehmet Özen, Celalettin Üstün, Bengi Öztürk, Pervin Topçuoğlu, Mutlu Arat, Mehmet Gündüz, Erden Atilla, Gülşen Bolat, Önder Arslan, Taner Demirer, Hamdi Akan, Osman İlhan, Meral Beksaç, Günhan Gürman, Muhit Özcan

**Affiliations:** 1 Ankara University Faculty of Medicine, Department of Hematology and Bone Marrow Transplantation Unit, Ankara, Turkey; 2 University of Minnesota, Department of Medicine, Division of Hematology-Oncology and Transplantation, Minneapolis, USA; 3 Ankara University Faculty of Medicine, Department of Internal Medicine, Ankara, Turkey; 4 Şişli Florence Nightingale Hospital, Clinic of Hematology, İstanbul, Turkey

**Keywords:** Chronic myeloid leukemia, Allogeneic transplantation, Tyrosine kinase inhibitors, Hematologic remission

## Abstract

**Objective:**

Tyrosine kinase inhibitors (TKIs) have changed the indications for allogeneic hematopoietic stem cell transplantation (allo-HSCT) in chronic myeloid leukemia (CML). Therefore, we aimed to evaluate the effect of TKIs on allo-HSCT in CML.

**Materials and Methods:**

In this quasi-experimental study, we compared patient, disease, and transplantation characteristics as well as allo-HSCT outcomes between the pre-TKI era (before 2002) and the post-TKI era (2002 and later) in patients with CML. A total of 193 allo-HSCTs were performed between 1989 and 2012.

**Results:**

Patients in the post-TKI era had more advanced disease (>chronic phase 1) at the time of transplant and more frequently received reduced-intensity conditioning compared to patients in the pre-TKI era. Relapse/progression occurred more frequently in the year ≥2002 group than in the year <2002 group (48% vs. 32% at 5 years, p=0.01); however, overall survival (OS) was similar in these two groups (5-year survival was 50.8% vs. 59.5%, respectively; p=0.3). TKIs (with donor lymphocyte infusions or alone) for treatment of relapse after allo-HSCT were available in the post-TKI era and were associated with improved OS. While the rates of hematologic remission at 3 months after allo-HSCT were similar between TKI eras, patients having remission had better disease-free survival (DFS) [relative risk (RR): 0.15, confidence interval (CI) 95%: 0.09-0.24, p<0.001] and OS (RR: 0.14, CI 95%: 0.09-0.23, p<0.001). Male allo-HSCT recipients had worse DFS (RR: 1.7, CI 95%: 1.2-2.5, p=0.007) and OS (RR: 1.7, CI 95%: 1.1-2.6, p=0.02) than females.

**Conclusion:**

TKIs are an effective option for the treatment of relapse after allo-HSCT in CML. Hematologic remission after allo-HSCT is also an important factor for survival in CML patients.

## Abstract (tr)

**Amaç:**

Tirozin kinaz inhibitörleri (TKİ) kronik myeloid lösemide (KML) allojenik hematopoetik kök hücre nakli (AHKHN) endikasyonlarını değiştirdi. Bu nedenle de biz KML’de TKİ’nin AHKHN üzerine etkisini değerlendirmeyi amaçladık.

**Gereç ve Yöntemler:**

Bu öncesi-sonrası çalışmasında KML’li hastalarda TKİ öncesi dönem (yıl <2002) ve TKİ sonrası dönem (yıl ≥2002) arasında hasta, hastalık ve nakil karakteristikleri ile AHKHN sonuçlarını kıyasladık. Toplamda 193 AHKHN, 1989 ve 2012 arasında yapıldı.

**Bulgular:**

TKİ sonrası dönemdeki hastalar, TKİ öncesi döneme göre nakil sırasında daha ileri hastalığa (kronik faz 1’den ileri) sahipti ve daha sık azaltılmış yoğunluklu hazırlık rejimi aldı. Relaps/progresyon ≥2002 grupta <2002 grubundan daha fazlaydı (5 yıllık %32’ye karşın %48; p=0,01); bununla birlikte toplam sağkalım (TS) bu iki grupta benzerdi (5-yıllık-sağkalım sırasıyla %50,8’e karşın %59,5; p=0,3). TKİ tedavisi (verici lenfosit infüzyonu ile birlikte veya tek başına) AHKHN sonrası relaps tedavisinde TKİ sonrası dönemde mevcuttu ve artmış TS ile ilişkiliydi. AHKHN sonrası 3. aydaki hematolojik remisyon oranları TKİ dönemleri arasında benzer iken; remisyonda olan hastalar daha iyi hastalıksız sağkalıma (HsS) [Göreceli risk (GR): 0,15; %95 güven aralığı (GA): 0,09-0,24; p<0,001] ve TS’ye sahipti (GR: 0,14; %95 GA: 0,09-0,23; p<0,001). Erkek AHKHN alıcılarının HsS (GR: 1,7; %95 GA: 1,2-2,5; p=0,007) ve TS’leri (GR: 1,7; %95 GA: 1,1-2,6; p=0,02) kadınlardan daha kötüydü.

**Sonuç:**

TKİ, KML’de AHKHN sonrası relapsın tedavisinde etkin bir seçenektir. AHKHN sonrası hematolojik remisyon da KML hastalarında sağkalımda önemli bir faktördür.

## INTRODUCTION

Chronic myeloid leukemia (CML) is a clonal disease that originates from a translocation between chromosomes 9 and 22 (Philadelphia chromosome). This translocation fuses ABL1 at 9q34 with BCR at 22q11.2, resulting in a chimeric gene that encodes an abnormal fusion protein. Before the discovery of tyrosine kinase inhibitors (TKIs), the median survival of CML patients in the blastic (BP), accelerated (AP), and chronic (CP) phases of disease who did not undergo transplant was 4-6 months, 1-1.5 years, and 3-8 years, respectively [1]. The only curative therapeutic option for CML was allogeneic hematopoietic stem cell transplantation (allo-HSCT), and all CML patients who had suitable human leukocyte antigen (HLA)-matched donors were considered candidates for allo-HSCT until 2002 [2].

It has been shown that imatinib treatment is superior to interferon alpha and low-dose cytarabine treatments in patients with CML [3], and later, TKI treatment was shown to result in long-term hematologic, cytogenetic, and molecular remission [4,5,6]. Therefore, the therapeutic landscape for CML has changed, and TKIs have become the first-line treatment for patients with CML. In 2002, TKIs became available for CML patients in Turkey [7,8]. Since 2002, allo-HSCT has remained the only proven curative option for CML, but it is currently indicated only for patients who have failed to respond to TKIs, those who have mutations associated with TKI resistance (e.g., T315I), and those who are intolerant to TKIs [8,9].

Although the discovery of TKIs has changed the indications for allo-HSCT in CML patients, allo-HSCT outcomes may have also been affected by the year of allo-HSCT due to the development of more successful transplantation techniques and supportive treatment options [10]. TKIs may also be used after allo-HSCT to treat relapse after transplantation in CML patients. Therefore, in this retrospective study, we compared allo-HSCT outcomes as well as patient, disease, and transplantation characteristics in the pre- and post-TKI eras and pretransplant TKI usage, posttransplant therapeutic TKI usage, and rates of reaching hematologic complete remission (CR) at 3 months in patients with CML.

## MATERIALS AND METHODS

We conducted this study after it was approved by the institutional ethics committee. A total of 188 CML patients underwent 193 allo-HSCTs (a second allo-HSCT was performed for five patients) at the Ankara University Department of Hematology and Bone Marrow Transplantation Unit between 1989 and 2012. CML clinical phases were defined according to the 2008 WHO criteria [11]. For this study, we defined the advanced phase as any phase other than CP1 (e.g., >chronic phase 1 (>CP1), AP, or BP). The majority of CML patients in the AP or BP of disease received acute myelogenous leukemia-type induction regimens before undergoing allo-HSCT.

We divided the patients into 2 groups: patients receiving allo-HSCT before TKIs were available (the pre-TKI era group, before 2002, n=128) and patients receiving allo-HSCT after TKIs were available (the post-TKI era group, 2002 and after, n=65) (Supplement 1).

In the post-TKI era, 48 of 65 patients (73%) received TKIs before allo-HSCT. We also evaluated these patients separately with regards to TKI effect on survival with two groups, a TKI-using group and a group not using TKIs, to differentiate the effect of pretransplant TKIs on allo-HSCT (Supplement 1).

Details about conditioning regimen, HLA matching status, graft-versus-host disease (GVHD) prophylaxis, definition and treatment of relapse, and supportive therapy are given in Supplement 1.

### Statistical Analysis

Numeric variables are presented as medians. Categorical variables were compared by the chi-square test or Fisher exact test. The nonparametric Mann-Whitney U test was used for noncategorical variables. GVHD was considered a categorical variable. Relapse and transplant-related mortality rates were calculated as time-dependent variables. Overall survival (OS) and disease-free survival (DFS) were calculated from the date of allo-HSCT. OS after relapse was calculated from the date of relapse. The distributions of OS and DFS durations in the two groups were estimated using the Kaplan-Meier method and compared using the log-rank test.

Recipient age, recipient sex, donor sex, stem cell source, conditioning regimen intensity, CML clinical phase, hematologic remission 3 months after allo-HSCT, and time from diagnosis to transplant were included in the multivariate analyses of survival. Logistical regression and a Cox model were used for risk factor analysis in DFS and OS. All reported p-values were two-sided, and p<0.05 was considered significant. Statistical analyses were performed using SPSS 16.0.

## RESULTS

Before 2002, allo-HSCT was performed in 321 patients, 128 of whom (40%) had CML. Between 2002 and 2006, allo-HSCT was performed in 581 patients, 65 of whom (11%) had CML (p<0.001). After 2007, the frequency of CML patients among all patients receiving allo-HSCT further decreased to 5% (n=18/360; p<0.001).

The time from diagnosis to transplantation was shorter in the pre-TKI era group than in the post-TKI era group (9.2 months versus 15.8 months, respectively; p<0.001, Table 1). The post-TKI era group more frequently underwent unrelated donor transplantation and received more peripheral blood stem cells (PBSCs); they also more frequently underwent reduced-intensity conditioning (RIC) and had more advanced disease (>CP1) (Table 1). Comparing patients for pretransplant TKI usage showed similar results. Patients using pretransplant TKIs had a longer time from diagnosis to transplantation, more unrelated donor transplantation, more received PBSCs, more RIC regimen, and more advanced disease than the group not using TKIs before transplant. The group not using TKIs before transplant also included younger patients than did the group using pretransplant TKIs (Table 1). Rates of reaching hematologic CR at 3 months after allo-HSCT were similar between the pre- and post-TKI eras and between the groups using and not using TKIs before transplant (Table 1).

Engraftment, rate of hematologic remission at 3 months after allo-HSCT, rate of sinusoidal obstruction syndrome of the liver, rate of acute and chronic GVHD, and rate of treatment-related mortality were similar in all groups. Hemorrhagic cystitis was more common in the pre-TKI era group (28.9%) than in the post-TKI era group (13.8%) (p=0.003) and more common in the group not using pretransplant TKIs (27.6%) than in the group using pretransplant TKIs (12.5%) (p=0.01) (Table 2).

### Outcomes

**Figure 1:**
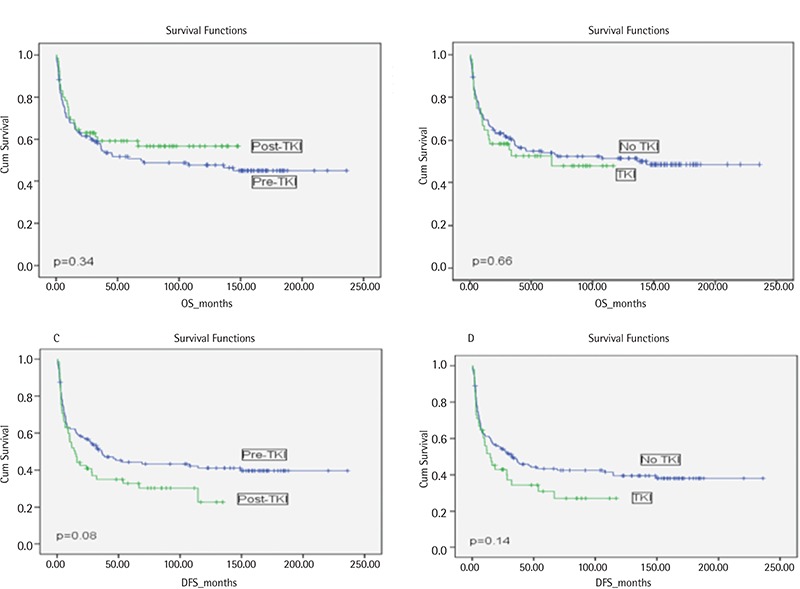
A. Overall survival in the tyrosine kinase inhibitor era. B. Overall survival in relation to tyrosine kinase inhibitor use. C. Disease-free survival in the tyrosine kinase inhibitor era. D. Disease-free survival in relation to tyrosine kinase inhibitor use.

**Figure 2:**
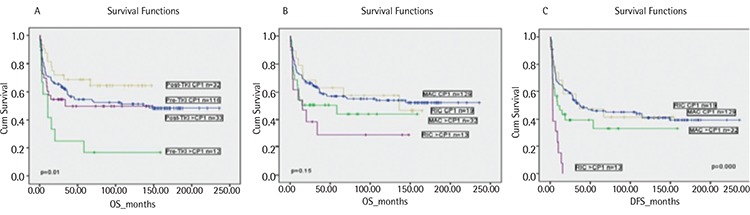
A) Overall survival by tyrosine kinase inhibitor era and phase of chronic myeloid leukemia. B) Overall survival by conditioning regimens and phase of chronic myeloid leukemia. C) Disease-free survival by conditioning regimens and phase of chronic myeloid leukemia.

Relapsed/refractory disease after allo-HSCT was observed in 57 patients (relapse in 49 patients and refractory disease in 8 patients). Relapse was more common in the post-TKI era group (Table 2). DFS and OS were similar between the pre- and post-TKI era groups and between the groups using and not using TKIs before transplant (Figure 1; Table 2). DFS and OS were also similar in CP1 and AP CML patients between the pre- and post-TKI era groups and between the groups using and not using TKIs before transplant (Table 2). However, pre-TKI patients with disease stage >CP1 had the worst OS rate, and this was significantly different from the OS rates of the other groups of patients (Figure 2).

OS after relapse in the post-TKI era group (mean: 94.2 months) was better than that in the pre-TKI era group (mean: 44.4 months). Patients received donor lymphocyte infusion (DLI) or TKIs, DLI plus TKI, or supportive therapy for the treatment of relapse. Most relapses (83%) in the pre-TKI era patients occurred before 2002, during which time TKIs were unavailable. The mean survival rates of patients receiving therapeutic TKI after relapse with DLI (86.8 months) and without DLI (95.5 months) were longer than those for patients receiving DLI alone (58.3 months). Patients who only received supportive treatment had the worst survival (6.5 months) (Table 3).

The median OS survival at 5 years after relapse was higher in the post-TKI era patients than in the pre-TKI era patients (respectively 67% vs. 28% in all patients, p=0.003; 83% vs. 32% in patients with CP1 CML, p=0.006; and 53% vs. 0% in patients with advanced disease, p=0.04) (data not shown).

Late relapses (9-12 years after allo-HSCT) occurred in 3 patients (one in the post-TKI era group and 2 in the pre-TKI era group). Two of these patients achieved CR with TKI treatment and survived. However, the third patient, who was diagnosed in the pre-TKI era, was resistant to TKI treatment and died.

In univariate analysis, recipient sex and phase of CML had a significant impact on DFS and OS (Table 4). Male recipients receiving grafts from female donors had the worst DFS and OS rates (Table 4), most likely because of the higher incidence of chronic GVHD in those patients (61% vs. 76% for sex-matched and mismatched conditions, respectively, p=0.03). The receipt of RIC regimens did not significantly affect OS but was associated with lower DFS in patients with advanced CML (Figure 2; Table 4). Although allo-HSCT from an unrelated donor was performed only in post-TKI era patients, donor type did not affect DFS or OS (Table 4). Additionally, the univariate analysis showed that TKI use and era of allo-HSCT did not affect OS or DFS (Tables 2 and 4; Figure 1). Hematologic CR at 3 months after allo-HSCT was also associated with better survival (Table 4).

In the multivariate analysis, male recipients (RR: 1.7, CI 95%: 1.2-2.5, p=0.007) and patients with advanced disease (RR: 1.8, CI 95%: 1.2-2.8, p=0.005) were associated with worse DFS. Male recipients (RR: 1.7, CI 95%: 1.1-2.6, p=0.02) were also associated with worse OS. However, advanced disease phase was not associated with worse OS. DFS and OS rates were similar between the pre- and post-TKI groups. In addition, hematologic CR at 3 months after allo-HSCT was associated with better DFS (RR: 0.15, CI 95%: 0.09-0.24, p<0.001) and OS (RR: 0.14, CI 95%: 0.09-0.23, p<0.001).

## DISCUSSION

In this study, we showed that the number of transplants for treatment of CML has significantly declined after TKIs became available (since 2002) and that allo-HSCT indications have changed. After TKIs, more CML patients receiving allo-HSCT had advanced disease. This might be important, especially for many developing countries that have limited beds and health care provider capacity to offer allo-HSCT. With this switch in allo-HSCT indication of CML, patients with more aggressive hematological malignancies can receive allo-HSCT in a more timely manner [16].

The clinical phase of CML is one of the most important factors affecting OS and DFS after allo-HSCT [17,18]. BP and AP patients had inferior outcomes to those of CP1 patients [17,19]. In a study performed during the TKI era, the outcomes of patients in CP1 were superior to the outcomes of patients in >CP1 [20]. Therefore, we defined patients with disease beyond CP1 as having advanced disease. However, we found that advanced disease only affects DFS, not OS. As expected, more patients with advanced disease (>CP1, AP, and BP) underwent allo-HSCT in the post-TKI era in our study. Despite the presence of more difficult cases in the post-TKI era, OS and DFS rates were similar in both eras. Due to the availability of first- and second-generation TKIs, allo-HSCT is performed late in most patients who require transplant; however, the difference between the groups for the time from diagnosis to HSCT was only approximately 6 months. This reasonably short delay may have contributed to the lack of inferior outcomes of patients in the post-TKI era.

Pretransplant TKI use did not affect OS or DFS in our study. A study by Khoury et al. [21] obtained similar findings, including no effect of pretransplant TKI use on OS. However, they did not provide the DFS rates of patients who received posttransplant TKIs. In our study, we showed that pretransplant TKI use did not affect DFS or OS in CML patients.

Jabbour et al. [22] showed that early complete response after TKI treatment in nontransplanted CML patients is the major determinant of patient outcome. Other studies reported that earlier and deep complete response after TKI treatment is correlated with better survival rates in CML patients [23,24]. However, to our knowledge, there is no study reporting the relationship between any response and overall survival in CML patients after allo-HSCT. Our study showed that early complete hematologic response after allo-HSCT is also one of the major determinants of CML patient survival.

The lower OS rates observed after relapse in pre-TKI era patients can easily be explained by the absence of TKIs. TKIs were available for posttransplant relapsed disease in the post-TKI era, and their use was associated with longer survival after CML relapse.

Savani et al. [25] reported that DLI with TKI treatment improved survival after allo-HSCT in 33 relapsing CML patients. They excluded BP CML patients within 30 days after DLI, but we included those patients in our study. Chalandon et al. [26] studied the occurrence of GVHD after DLI in CML patients and found that GVHD-related DLI was associated with mortality. Although we did not evaluate the occurrence of GVHD after DLI, we found that after allo-HSCT, TKI treatment (with or without DLI) for CML resulted in longer OS compared to the DLI-only approach. This finding explains the central role of TKIs in the treatment of relapse after allo-HSCT. Improvements in supportive measurements and other developments in the field most likely also contributed to the improved survival in the post-TKI era. Allo-HSCT outcomes of our CML patients may have also been affected by the year of allo-HSCT due to the development of more successful transplantation techniques and supportive treatment options [10].

OS was inferior in male recipients, in particular those who received grafts from female donors. Therefore, male recipients with female donors had the worst OS, most likely due to a higher incidence of chronic GVHD. When excluding male recipients receiving grafts from female donors, there were no significant differences between the OS or DFS rates among the other three groups.

The complications of allo-HSCT were similar in the pre-TKI and post-TKI era groups, except in regards to hemorrhagic cystitis. Hemorrhagic cystitis occurred more frequently in the pre-TKI era, possibly due to the greater frequency of myeloablative conditioning regimen use during that period [27].

We found that pretransplant administration of TKIs has no negative impact on engraftment. Furthermore, we considered the fact that most of the patients in the post-TKI era group were challenged with an important drug, a TKI. This may have created clinically or biologically difficult cases, as observed in lymphoma patients with disease relapse shortly after being treated with chemotherapy regimens containing rituximab [28].

## CONCLUSION

In conclusion, as expected, the frequency of allo-HSCT for CML patients sharply decreased after the introduction of TKIs. In recent years, this rate slightly increased, most likely due to TKI failure. Although CML patients who underwent allo-HSCT in the post-TKI era had more advanced disease, early and late outcomes were comparable between the pre- and post-TKI eras, mostly due to the high efficiency of TKIs for the treatment of relapses after allo-HSCT and advancements in the stem cell transplantation field. In addition, CR after allo-HSCT has improved survival rates and is the most prominent factor affecting OS and DFS.

##

**Supplement 1. TKI treatment before allogeneic hematopoietic stem cell transplantation.**

From 2002 to 2006, although TKIs were available for clinical use, their long-term effects were unknown; therefore, allo-HSCT was performed for all CML patients during that time period. After 2006, allo-HSCT was mainly considered for TKI-resistant/intolerant CML patients or CML patients in advanced phases of the disease. In the post-TKI era, allo-HSCT was performed in 65 CML patients: 6 patients in BP, 5 patients in AP, 22 patients in the second chronic phase (CP2), and 32 patients in the first chronic phase (CP1) (7 patients were resistant/intolerant to TKIs, 12 patients were sensitive to TKIs, and 13 patients did not receive TKIs based on the physician’s or patient’s preference due to the lack of knowledge regarding their long-term effects).

Most patients using TKIs before allo-HSCT were treated with imatinib alone (n=41), 4 patients received both imatinib and dasatinib, and 3 patients were treated with imatinib, dasatinib, and nilotinib before transplantation. Mutational analysis was performed for 9 patients, and only one patient was positive for the T315I mutation.

**Conditioning Regimen**

The most frequently used myeloablative conditioning (MAC) regimen contained combined cyclophosphamide (CY) (120 mg/kg i.v.) and busulfan (3.2 mg/kg i.v. or 4 mg/kg p.o., 4 days) treatment with or without antithymocyte globulin (ATG) (10 mg/kg/day, 4 days) and combined CY (120 mg/kg) and fractionated total-body irradiation (12 Gy) treatment with or without ATG (10 mg/kg/day, 4 days).

Fludarabine-based regimens have been used as RIC regimens: combined fludarabine (30 mg/m^2^ i.v., 6 days) and busulfan (3.2 mg/kg i.v. or 4 mg/kg p.o., 2 days) treatment with or without ATG (10 mg/kg/day, 4 days) or combined fludarabine (30 mg/m2, 6 days) and cytarabine (3 g/m^2^ b.i.d., 4 days) treatment with or without ATG (10 mg/kg/day, 4 days). We did not perform in vitro T-cell depletion; however, in vivo T-cell depletion was accomplished by ATG administration in cases of a mismatched and/or unrelated donor (URD) after both MAC and RIC conditioning regimens.

**HLA Matching Status**

HLA matching status was defined as follows: well matched if recipient/donor pairs had either no identified HLA mismatches and informative data for at least 6 loci or matching alleles at HLA-A, -B, and -DRB1; partially matched if recipient/donor pairs had a defined, single-locus mismatch and/or missing HLA data; and mismatched if recipient/donor pairs had ≥2 allele or antigen mismatches [12,13]. URD was started at our institution after 2002 for patients who had no HLA-matched donor or related donor with 1 allele mismatched and HLA match statuses were studied for URD transplants with at least 10 loci or alleles including HLA-A, -B, -C, -DQ, and -DRB1. After 1998, RIC regimens were administered to 21 patients due to either advanced age (≥50 years) or comorbidity. Eleven patients received RIC transplant in a clinical trial comparing the intensity of conditioning regimens in CP CML patients.

**GVHD Prophylaxis**

GVHD prophylaxis consisted of methotrexate (Mtx) at 15 mg/m^2^ on day +1 and 10 mg/m^2^ on days +3 and +6 (and additionally on day +11 for unrelated donor allo-HSCT) and daily cyclosporine (CSA) from day -1 (or -3 for unrelated donor allo-HSCT) to day +180.

**Defining and Treating Relapse**

Relapse after allo-HSCT was defined by molecular, cytogenetic, or hematologic findings. Between 1989 and 1999, patients were followed cytogenetically, and molecular evaluation was not the main technique for remission assessment of CML patients. By 1999, molecular techniques were primarily used in place of cytogenetic techniques. Both molecular and cytogenetic data after allo-HSCT were only available after 1999; thus, these data were not included in the study. After 1999, patients were followed molecularly by testing BCR-ABL transcripts in RNA samples of peripheral blood or bone marrow starting at the time of allo-HSCT using a RQ-PCR method (T922, LightCycler Quantification, Roche Diagnostics, Munich, Germany). The molecular methods for *BCR-ABL1* and chimerism studies were performed every 3 months until 1 year, every 6 months until 5 years, and every 1 year until 10 years after allo-HSCT. Logarithmically increasing levels of *BCR-ABL* transcript levels in at least 2 consecutive tests were defined as molecular relapse. Hematologic complete remission was defined as the detection of leukocytes at <10,000/µL, platelets at <450,000/µL, and basophils at <5%; the absence of myeloblasts, myelocytes, and promyelocytes in peripheral blood; myeloblasts at <5% in bone marrow; and the absence of a palpable spleen on physical examination.

Chimerism was analyzed by PCR-based amplification of short tandem repeats (3130 Genetic Analyzer, Applied Biosystems, Foster City, CA, USA). An increase in recipient signals of more than 5% in sequential estimations of molecular chimerism compared to the prior level was considered as graft failure. In patients with graft failure or molecular/hematologic relapse, escalated doses of donor lymphocyte infusions (CD3+ cells in 1x10^7^, 5x10^7^, and 1x10^8^ doses) were administered sequentially each month (if no response had been observed and no GVHD had developed since the previous infusion) and/or TKIs as a therapeutic option were administered.

**Supportive Therapy**

Prophylactic platelet transfusion was given if the platelet count was <20x10^9^/L. Red blood cell transfusion was given if hemoglobin was <7 g/dL or <10 g/dL depending on the patient’s history of cardiovascular events. All blood products were irradiated and filtered. Infection prophylaxis has not changed since 1988 and includes acyclovir, fluconazole, and trimethoprim/sulfamethoxazole for all patients. Ciprofloxacin was added to these antimicrobials for patients receiving MAC. All patients were treated following the guidelines reported by the Infectious Diseases Society of America [14] and the Turkish National Febrile Neutropenia Study Group [15].

## Figures and Tables

**Table 1:**
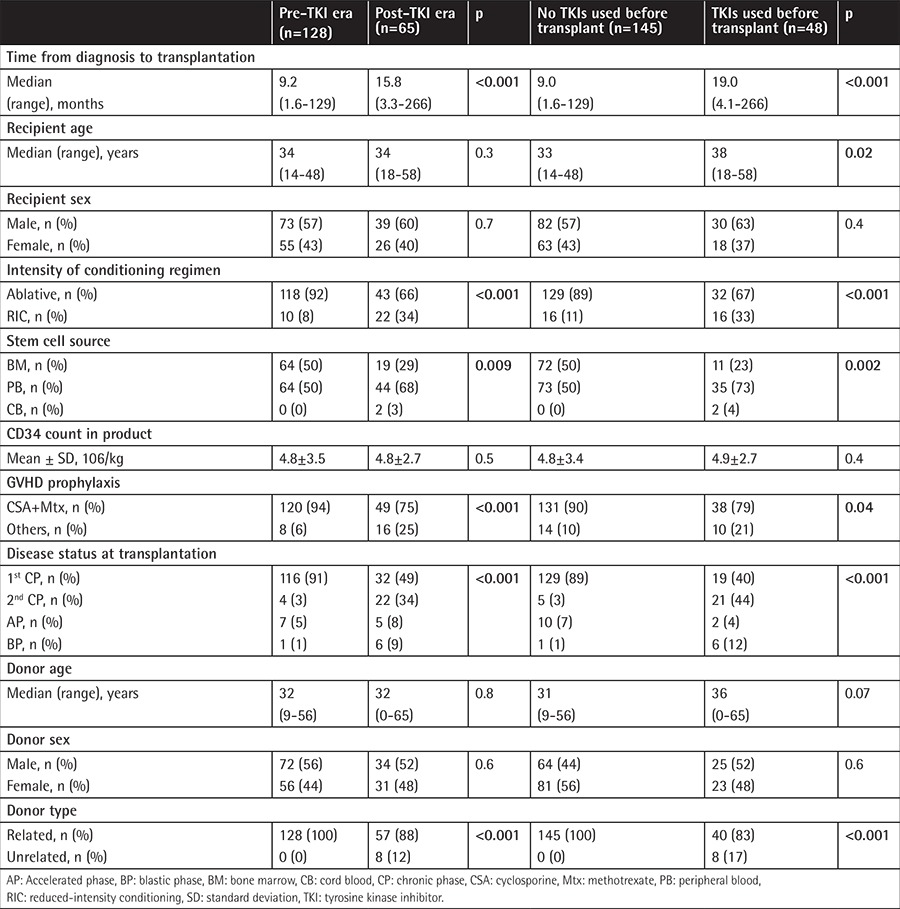
Patient and transplantation characteristics in the pre-tyrosine kinase inhibitor and post-tyrosine kinase inhibitor eras with and without pretransplant tyrosine kinase inhibitor usage.

**Table 2:**
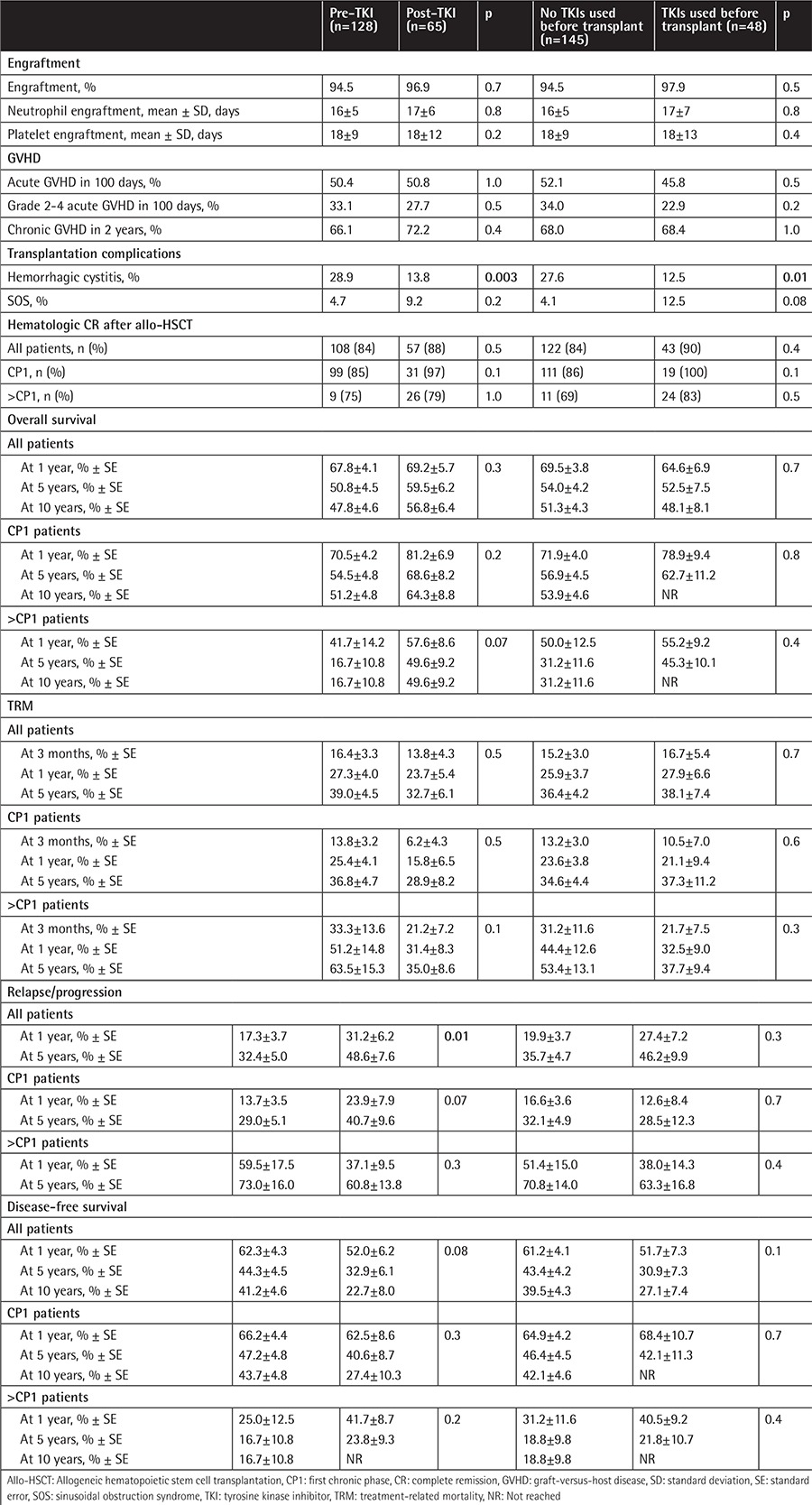
Outcomes of transplantation regarding tyrosine kinase inhibitor era and tyrosine kinase inhibitor use.

**Table 3:**
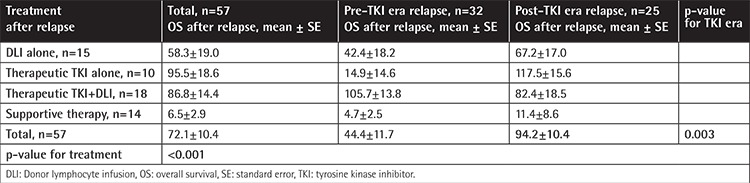
Treatment and survival after relapse.

**Table 4:**
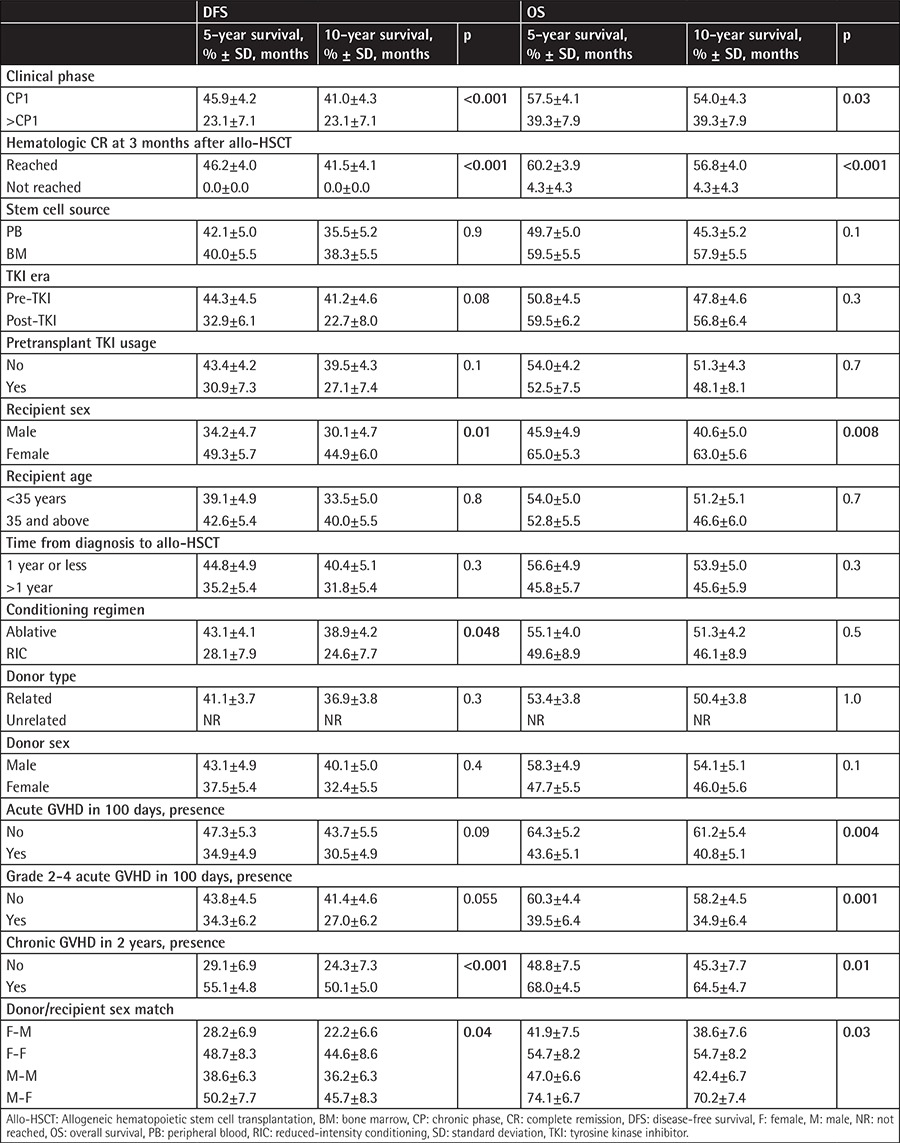
Univariate analysis for all patients for disease-free survival and overall survival.
